# A morphometric approach to track opium poppy domestication

**DOI:** 10.1038/s41598-021-88964-4

**Published:** 2021-05-07

**Authors:** Ana Jesus, Vincent Bonhomme, Allowen Evin, Sarah Ivorra, Raül Soteras, Aurélie Salavert, Ferran Antolín, Laurent Bouby

**Affiliations:** 1 Integrative Prehistory and Archaeological Science (IPAS), Universität Basel, Basel, Switzerland; 2 ISEM, University of Montpellier, CNRS-IRD-EPHE, Montpellier, France; 3 Archéozoologie, Archéobotanique: Sociétés, Pratiques et Environnements (AASPE), Muséum National d’Histoire Naturelle, CNRS, Paris, France; 4 Department of Natural Sciences, German Archaeological Institute (DAI), Berlin, Germany

**Keywords:** Plant domestication, Archaeology, Taxonomy

## Abstract

Opium poppy (*Papaver somniferum* L. subsp*. somniferum*) was likely domesticated in the Western Mediterranean, where its putative wild ancestor is indigenous, and then spread to central and northern Europe. While opium poppy seeds are regularly identified in archaeobotanical studies, the absence of morphological criteria to distinguish the seeds of wild and domestic forms prevents the documentation of their respective historical and geographical occurrences and of the process of opium domestication as a whole. To fill this gap and better understand the status of this crop in the Neolithic, we combined seed outline analyses, namely elliptic Fourier transforms, with other morphometric descriptors to describe and identify *Papaver setigerum, Papaver somniferum* and other *Papaver* taxa. The combination of all measured parameters gives the most precise predictions for the identification of all seven taxa. We finally provide a case study on a Neolithic assemblage from a pile-dwelling site in Switzerland (Zurich-Parkhaus Opéra, ca. 3170 BC). Our results indicate the presence of mixed populations of domestic and wild seeds belonging to the *P. somniferum* group, suggesting that the plant was already in the process of domestication at the end of 4th millennium BC. Altogether, these results pave the way to understand the geography and history of the poppy domestication and its spread into Europe.

## Introduction

Opium poppy (*Papaver somniferum* L.), as the principal source of opium and opiate drugs, today, as in the past, is a most controversial species. This plant has multiple uses including medicine (e.g. morphine), decoration (as an ornamental plant) and food. Poppy seeds can be used for making porridge, eaten raw or pressed for edible oil^1^. Unlike the founder crops (different cereals, pulses and flax) that are known in Europe, arriving from the Near-East during the Neolithic period (ca. 6500–3500 BC), opium poppy is currently supposed to have been domesticated outside of the Fertile Crescent. Its domestication probably took place in the Western Mediterranean area from where the putative progenitor is native and still growing wild today, *Papaver* *somniferum* subsp. *setigerum* (DC.) Arcang.^2–6^ (from now on *P. setigerum*). *Papaver somniferum/setigerum* seeds are reported in the archaeological record starting from the Neolithic period (6th-millennium cal. BC)^5,7,8^. Regrettably, these are not identified to subspecies/status level (i.e. at the wild/domesticated level) because no clear criterion exists for these seeds to be distinguished. This paper aims to fill this methodological gap to further gain knowledge for the archaeological and the botanical sides of *Papaver* domestication history. The goals of the paper are to distinguish the wild from domestic species in modern *Papaver* through the application of traditional and geometric morphometrics on seeds. Then we use this methodology to establish the status of this plant during the Neolithic period using archaeological seeds from a case study in central Europe, Zurich-Parkhaus Opéra (ca. 3170 BC). This is the first time this approach is used to study the domestication process of opium poppy.

The genus *Papaver* encompasses more than 80 different species^9^ of annual, biennial and perennial plants distributed in central and south-western Asia, central and southern Europe and northern Africa^10^. All species of *Papaver* grow in open and unevenly disturbed habitats. Perennials and biennials are mountain taxa growing above 1000 m while annuals are mostly lowland taxa^11^. *Papaver* species encountered in western and southern Europe and identified in the archaeological record are: *P. album*; *P*. *hybridum*; *P. rhoeas*; *P. argemone;* as well as different subspecies and one variety of the *P. somniferum* group (here referred at the species level for the sake of simplicity): *P. somniferum*, *P. setigerum* and *P. nigrum*.

*Papaver* taxonomy is still debated, Kadereit^10^, Zohary et al*.*^6^ and Carolan et al*.*^12^ all argued that *P*. *somniferum* has two subspecies: *setigerum* and *somniferum,* the latter being the domesticated descendant. Whether *P. setigerum* and *P. somniferum* represent two distinct species or whether they should be considered as two subspecies is still debated. Using sequences of the plastid gene rpl16 and the rpl16-rpl14 Hosokawa et al*.*^13^ argued that both species were identical. Likewise, a phylogenetic study of *Papaver* based on DNA sequences was unable to distinguish these two taxa^12^. The opium poppy (*P*. *somniferum*) is an annual herb, 30–150 cm high, self-pollinated and most of the actual cultivars are diploid. *Papaver setigerum* is an annual plant, 60 cm high, a field weed occurring in disturbed grounds^14^ and native to the western Mediterranean^6,14^ in Algeria, France, Italy, Morocco, Portugal, Spain, Tunisia^15^. *P*. *setigerum* is both diploid and tetraploid and inter-fertile with the *P. somniferum* cultivars^16^.

The history and mechanisms of opium poppy domestication remain unclear despite the abundance of archaeological seeds in sites dated to the Neolithic period, particularly in the Alpine Foreland^5^. Archaeobotanical remains are usually broadly identified as *P. somniferum*, yet their domesticated status is unclear^7^. The domestication syndrome of opium poppy encompasses the increase in the size of the capsule and seeds, as well as capsule indehiscence^6^. Previous studies attempted to distinguish wild from domestic opium poppy seeds based on the size, comparing archaeological seeds to modern species^17–22^. However, the size of the seed alone has not proven to be a good discriminating criterium^20^ since it overlaps between the two species.

This paper addresses two questions: (i) can we distinguish between modern seeds of the wild (*Papaver setigerum*)*,* domestic (*P. somniferum)* and other *Papaver* species? If so, (ii) can we distinguish *Papaver* species in archaeological assemblages previously identified as *P. setigerum/somniferum*?

The modern plant material consisted of 270 seeds belonging to seven *Papaver* taxa (30 seeds per taxon) obtained from the seed collection of the Integrative Prähistorische und Naturwissenschaftliche Archäologie (IPNA/IPAS) at the University of Basel, Switzerland (Supplementary Material Table 1). Two additional sets of 30 seeds of *P. somniferum* and *P. setigerum* were obtained from the Graineterie (seed collection) of the National Museum of Natural History (MNHN) in Paris, France. We first established new identification criteria between *Papaver* species, and chiefly between *P. setigerum* and *P. somniferum*. We applied traditional and geometric morphometrics on seeds, considering the number of cells, size measurements and shape using outline analysis. Outline analysis has been successfully used to identify archaeobotanical remains of an array of species such as grape pips^23^, olive stones^24^ cereals^25–27^, dates^28^ and cherry stones^29^. The technical challenge for *Papaver* seed lies in the millimetric size of the seeds and their globoid shape. Prior to any morphometric analysis, repeatability tests were performed to establish the effects of taking the photos, cleaning and landmarking in the observed seed morphometric variation.

**Table 1:** Results of the reproducibility tests (seed positioning, photography cleaning and landmarking) performed through Anova: F: Fisher statistics, *P*-value and measuring error (%) when comparing the three taxa of *P. somniferum*.

Taxa	ANOVA F	*P*-value	%ME
**Position test**			
*P. somniferum*	1.3208	0.093	85.339
*P. setigerum*	2.3065	0.039	79.283
*P. nigrum*	3.2988	0.013	68.504
**Cleaning test**			
*P. somniferum*	4.4525	0.004	46.493
*P. setigerum*	17.502	0.001	15.382
*P. nigrum*	7.079	0.001	33.043
**Landmark test**			
*P. somniferum*	37.649	0.001	7.566
*P. setigerum*	13.648	0.001	19.171
*P. nigrum*	27.335	0.001	10.226

This protocol was then applied to 33 uncharred poppy seeds preserved in waterlogging conditions from a Neolithic pile-dwelling site in the Alpine Foreland (Zurich-Parkhaus Opéra, dendro-dated to ca. 3170 BC^30^). This site is an ideal starting point since the Swiss Plateau is outside of the natural area of spread of *P*. *setigerum*, thus suggesting a human introduction. *P*. *somniferum* seeds are known in Switzerland since ca. 5000–4800 BC in the Valais region^31^, and seed and capsule fragments were recovered in large quantities in pile-dwelling sites starting from 4300 BC, indicating widespread cultivation^32^. Zurich-Parkhaus Opéra is, therefore, a perfect case study to test our methodology, since opium poppy had been cultivated in the area for ca. 1000 years and might therefore show morphometrical signs of domestication. Furthermore, the waterlogging conditions maintained the original seed shape and size, unlike what is known to occur to charred remains^33^.

## Results

### Measurement error

The error measurements was quantified by acquiring data 3 times on 5 seeds independently for the three species (Table 1), which allow to test for the different steps of the protocol: positioning, image cleaning and landmarking. These three steps yield contrasting results. Positioning error is high (between 68 and 85%) (Table 1). This originates from the difficulty to orientate the seed consistently under the stereomicroscope, due to the small size and globoid shape of poppy seeds. On the other hand, cleaning and landmarking errors are much lower. Despite the existence of a certain positioning error, this does not prevent taxonomic identification (see below) and the protocol can therefore be used for the purpose of this study.

### Phenotypic variation among species

**Figure 1:**
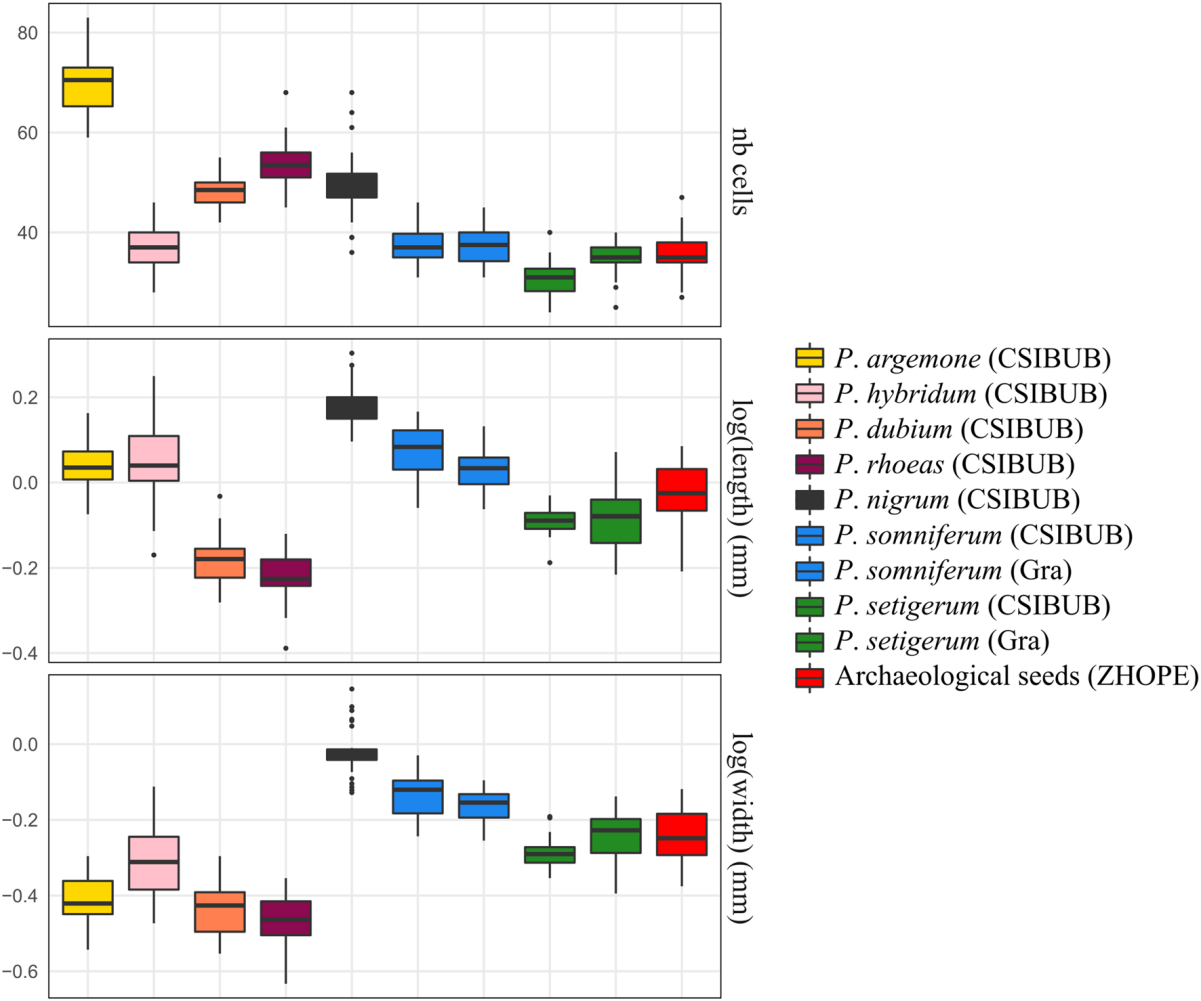
Boxplot of measurements (number of cells, length, width,) made on the modern seeds. Length and width are (natural) logged. Archaeological seeds are presented in red.

Length and width show considerable variation with significant differences between the various *Papaver* species (Fig. 1), as reported by the results of the Kruskal–Wallis tests (length: χ^2^ = 231.78, df = 7, *P* < 10^–16^; width: χ^2^ = 243.76, df = 7, *P* < 10^–16^). The two domestic species (*P. somniferum* and *P. nigrum*) have bigger seeds than the wild species, especially in width (Wilcoxon rank tests, all P values < 10^–11^). The seeds of *P. somniferum* and *P. setigerum* are different in size (Wilcoxon rank tests, all *P* values < 10^–8^). For both species, the two investigated samples are close, yet some dimensions appear different (Wilcoxon rank tests, *P. setigerum*, width *P* = 0.003; *P. somniferum* length *P* = 0.005). The number of cells also present differences between species (Fig. 1, Kruskal–Wallis tests number cells: χ^2^ = 233.21, df = 7, *P* < 10^–16^) and between samples of *P. setigerum*. *P. argemone* is clearly the species with more cells. *P. nigrum* is also different from the other species of the *P. somniferum* group. *P somniferum* and *P. setigerum* and consequently the archaeological seeds are very close regarding this criterion.

**Figure 2:**
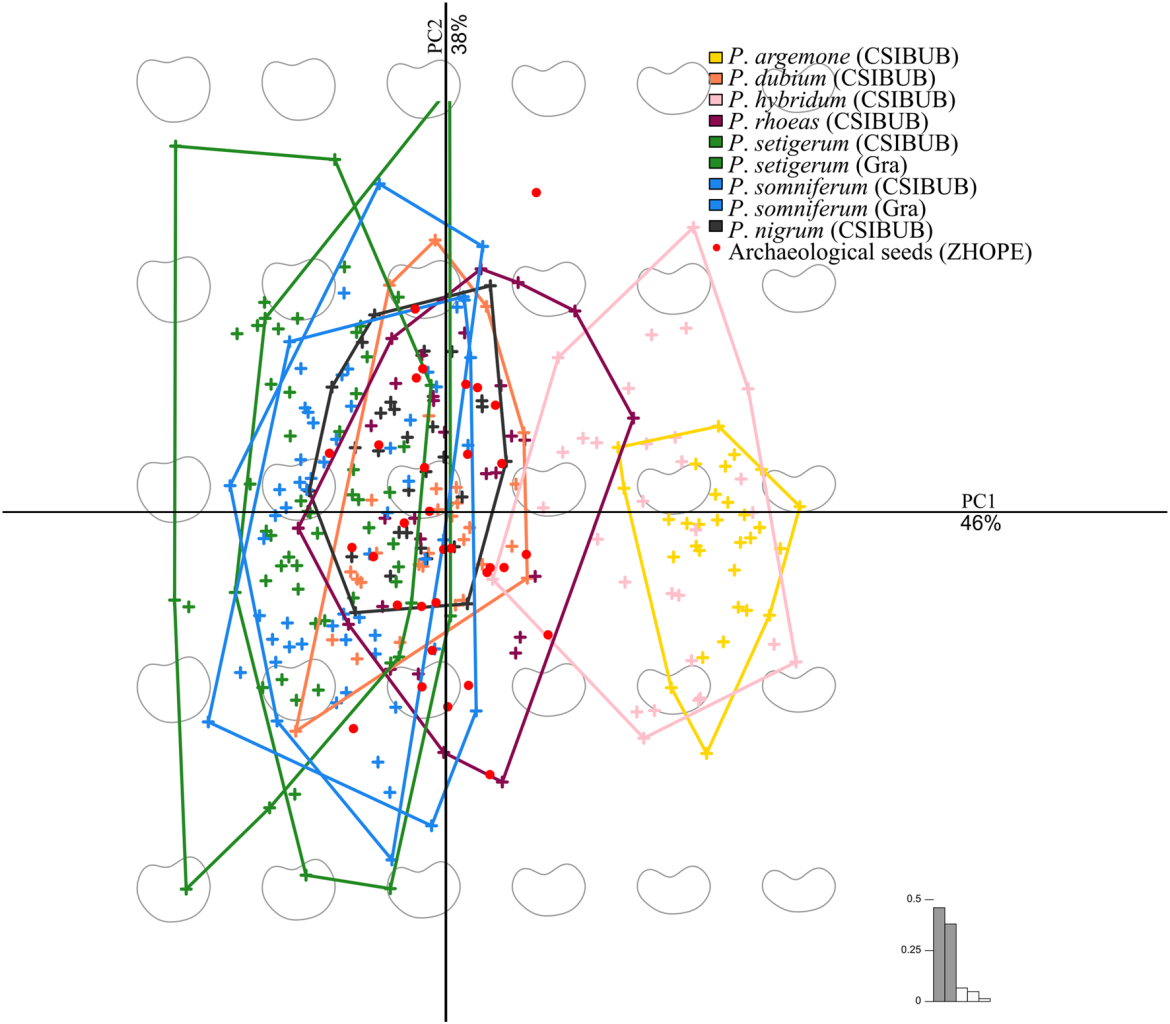
Principal component analysis performed on shape coefficients. The first two components are shown here gather 84% of the total shape variability. Archaeological seeds (red dots) are added as supplementary individuals, i.e. reprojected, on this biplot.

The first two PCs (Fig. 2) gathered 84% of the total shape variation. Shape changes along PC1 (46%) are related to roundness while changes along PC2 (38%) correspond to an asymmetry component between the two parts of the seed. Asymmetry mostly represents intraspecific variability. It is higher for the species with the most rounded seeds (*P. setigerum* and *P. somniferum*).

The permutational MANOVA (df = 6, F = 32.126, adj. r^2^ = 0.42, *P* = 0.001) showed differences in shape between taxa. The species with the most elongated seeds (*P. argemone* and *P. hybridum*) are clearly distinguished from the other species with proportionally rounder seeds. Shape overlapping is particularly important between *P. setigerum*, *P. somniferum*, *P. nigrum* both on PC1 and PC2.

**Figure 3:**
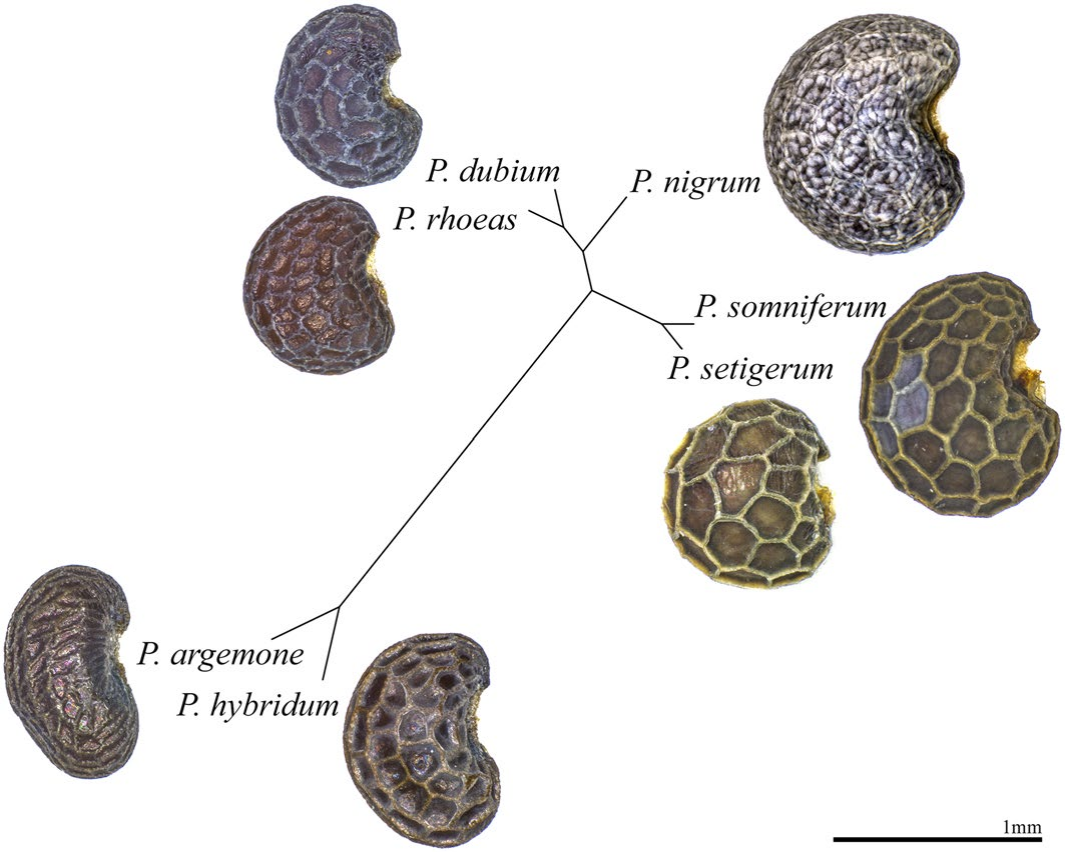
The unrooted tree obtained with hierarchical clustering on the Euclidean distance matrix between Fourier coefficients averaged per taxa.

**Figure 4:**
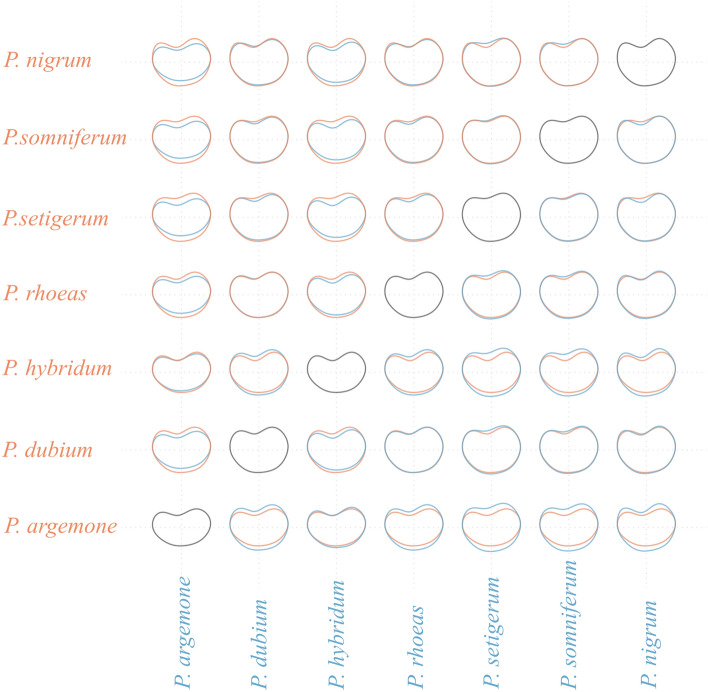
Mean shapes pairwise comparisons for all *Papaver* taxa studied here. Orange colour corresponds to the taxon of the rows and the blue colour to the taxon of the columns.

The hierarchical clustering performed on the euclidean distance matrix computed on the coefficients averaged per taxa confirmed the shape proximity between *P. setigerum* and *P. somniferum*, as well as between *P. rhoeas* and *P*. *dubium* and, in the other branch, *P*. *hybridum* and *P*. *argemone*. (Fig. 3). The slight differences in shape between the species of the *P. somniferum* group occur in part surrounding the hilum (Fig. 4 and Fig. 1 in Supplementary material).

### Identification of modern seeds

**Figure 5:**
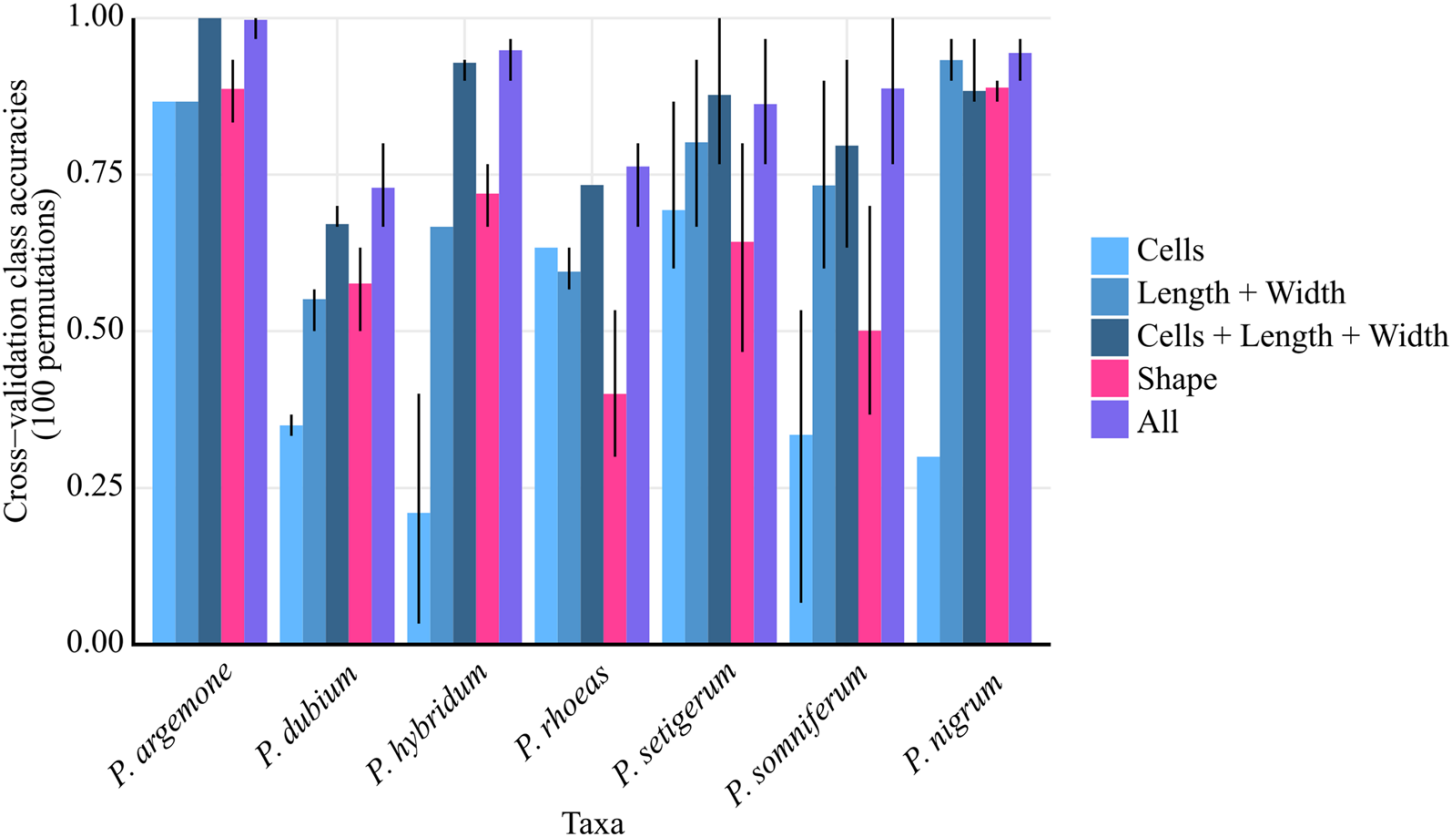
Benchmarking of linear discriminant analyses on all species and using different proxies. Accuracy per classes and their variability were obtained using 100 permutations on classes-balanced dataset with the error bars.

The linear discriminant analyses (LDA) on modern material allowed a good identification at the species level. The percentage of accuracy identification using the cells and size ranged between 67 and 73% for two taxa (*P. dubium* and *P. rhoeas*) but for the other five taxa it was above 80% (Fig. 5).

Although the performance of individual variables (length, width, shape and cell number) provided relatively good discrimination, the best percentages were obtained when all traditional and shape parameters were combined (Fig. 5: 73–100%).

The results were similar if we considered only the species from the *P. somniferum* group with two taxa and three taxa (Figs. 2 and 3 in supplementary material). Combining all variables, more than 87% of the seeds were correctly identified to their specific taxon (Supplementary data Fig. 2).

### Assignation of archaeological seeds

**Figure 6:**
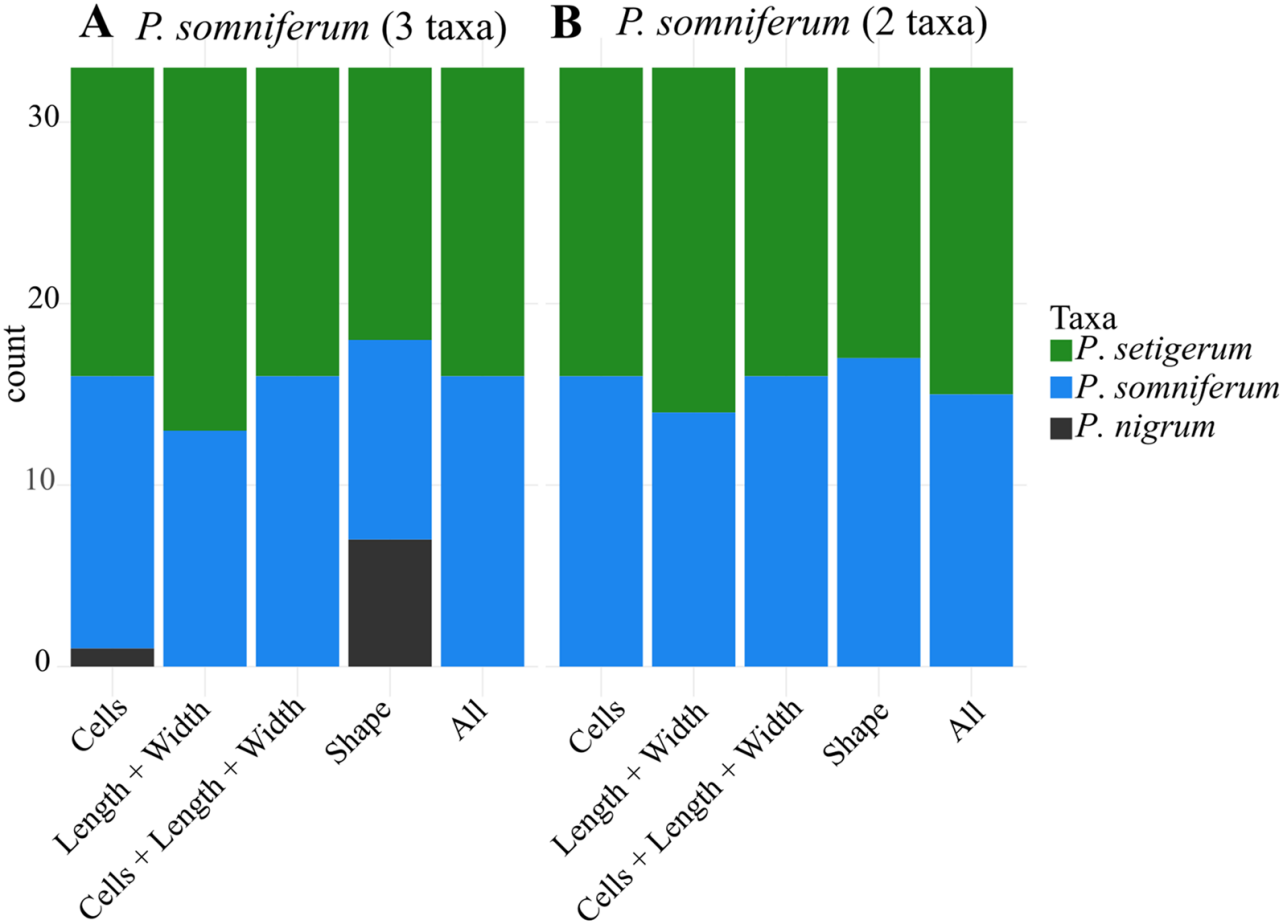
Assignation of the 33 archaeological seeds: Benchmarking of linear discriminant analyses on (A) three taxa (*P. setigerum*, *P. somniferum* and *P. nigrum*) and (B) two taxa (*P. setigerum*, *P. somniferum*) using different variables or set of variables.

Length and width showed considerable variation between modern seeds of *Papaver* and archaeological seeds (Fig. 1). The width of the archaeological seeds of Zurich-Parkhaus Opéra is closer to modern *P. setigerum* seeds while the length is intermediate between *P. setigerum* and *P. somniferum* modern seeds (Fig. 1). The LDAs using various sets of descriptors were used to infer species on the archaeological material. The archaeological seeds from Zurich-Parkhaus Opéra were identified as *P. setigerum* and *P. somniferum* (see Fig. 6A)*.* Some seeds were identified as *P. nigrum* only when using one of the descriptors: number of cells (3%) and shape (21%). When all criteria are combined, no seed is allocated to *P. nigrum*. In every case, and more especially when all criteria are combined, about half of the seeds are attributed to *P. setigerum* and a half to *P. somniferum*.

## Discussion

Here we show that the combined application of morphometric descriptors, number of cells and shape analysis outline elliptic Fourier transforms (EFT) allows the discrimination of seven modern species of *Papaver* genus.

In spite of a high positioning error, due to the small size of this material, morphometrics can be done. The various species and sub-species are well discriminated, which validates the methodology used (Table 1). Indeed, the most interesting result was that by using this method, with all descriptors, the LDA gave optimal results when distinguishing between *P. setigerum* and *P. somniferum* as well as when compared to other *Papaver* related modern seed species. The second finding of this study was that allow for the first time the application of this method to archaeological seeds.

### Phenotypic variation among modern seeds

The first question that this study sought to answer was if it is possible to discriminate between the different taxa. The LDA results (Fig. 5) show that it is possible to distinguish the taxa with high accuracy results by using all the descriptors or the combination of the number of cells and size.

According to our results, adding the cell number to the size descriptors gives a better prediction for *P. dubium*, *P. rhoeas* and *P. somniferum* (Fig. 5). These are also the taxa where the shape yielded the lowest additional accuracy. A possible explanation for this confusion might be that their shape is identically reniform (as seen in Fig. 4). Instead, shape is a powerful descriptor in the case of *P. nigrum, P. argemone* and *P*. *hybridum* seeds, which are more elongated, with the accuracy being over 93% (Fig. 4).

The results of the unrooted distance network (Fig. 3) showed morphometrics proximities that mirror the results of previous phylogenetic studies^12^. On the one hand, Carolan et al*.*^12^ identified *P. setigerum* and *P. somniferum* as sister group with a common ancestor^12^. On the other hand, *P. hybridum* and *P. argemone* also have a common ancestor, which again is congruent with our results based on seed shape (Fig. 3).

Our results show that cell number alone gives lower accuracy (23–33% see Fig. 5). Surprisingly, it is a critical discriminant variable in the identification of *P. setigerum* (with the lowest number of cells) and *P*. *argemone* (with the largest number of cells). The importance of cell number, as well as cell size, were also the previous observed^18,20,21^, however, according to our results the total number of cells from one of the seed face gives better discrimination results than solely count the cells by rows or measuring the cells^18,20,21^.

### P. somniferum seeds

The results show that it is possible not only to distinguish *P. somniferum* from the other taxa but also between *P. somniferum* subspecies (Figs. 2 and 3 in supplementary material). Previous studies on the *Papaver* genus encountered problems distinguishing mainly *P. somiferum* apart from *P. setigerum*^20^*.* Based mainly on size, other authors^16,20^ stated that the high variability within the species *P. somniferum* makes it impossible to distinguish between the different subspecies. Future research may include a higher number of varieties and accessions to better assess the intra(sub)specific variability in terms of cell number, size and shape, including in terms of environmental conditions^34^.

### Assignation of archaeological seeds

The classification of archaeological seeds from the late Neolithic site of Zurich-Parkhaus Opéra (Switzerland) based on the model trained on modern seeds allow for the first time to apply this method to these small seeds (Fig. 6). In this case, it is essential to combine all descriptors and not only the shape. The results of the taxonomic attribution of the archaeological seeds found in Zurich-Parkhaus Opéra suggest a mixed population of domestic and wild-type seeds. Nevertheless, one should consider the caveats of using modern material to classify archaeological specimens and of the preservation of archaeological remains. Swelling of the seeds in waterlogged preservation^1,18^ may also play a role in the model’s prediction. In order to develop an understanding of the possible effects of taphonomic factors in the future, two tasks should be implemented. One would be to increase the number of samples, and the other one should be to perform experiments to replicate the state of the archaeological seeds. Nevertheless, the archaeological material results suggest that well-preserved waterlogged seeds of *Papaver* species can be used for this type of analysis.

In our results, 18 seeds were attributed to *P. setigerum* and 15 seeds to *P. somniferum*. This may be interpreted in two ways: the population at Zurich-Parkhaus Opéra is a mixture of wild and domestic forms belonging to a population in an intermediate stage of domestication, or we have a fully domestic form with some wild individuals still surviving as weeds in the fields. This opens several scenarios to explain the process of domestication of poppy. There is evidence of the use and potential cultivation of opium poppy in the Western Mediterranean since ca. 5600 BC, according to the finds at the lake village of La Marmotta, Italy (ca.5620–5480 Cal BCE)^8,35^ and in several other sites, for instance, at the pile-dwelling site of La Draga, Spain, ca. 5200 BC^3,8^. The authors of both archaeobotanical studies actually suggest that opium poppy was cultivated, based on the number of finds and their ubiquity^3,35^. Nevertheless, it is possible that the plant was still morphologically wild since these sites fall within the native area of *P. setigerum* and isolation of the cultivated population would have been more difficult. Thus, it is unclear if opium poppy spread northwards as morphologically wild, not fully domesticated, or as a domesticated plant. It is actually possible that opium poppy arrived at the Alpine foreland as a domesticated form along with some wild *P. setigerum* forms as weeds.

The domestication process could have been accelerated with the beginning of cultivation of opium poppy outside of the area of the natural distribution of the wild subspecies such as in the Alpine Foreland around 4300 BC^32^. After ca. 1100–1200 years of cultivation of opium poppy in this region, the Zurich-Parkhaus Opéra seeds seem to indicate that the plant is still in the process of being domesticated. This may be interpreted as an indication of a protracted domestication process^36^, as observed with other plants domesticated in the Neolithic period. In order to test this hypothesis, similar analyses should be performed on opium poppy seeds from archaeological sites located in the Western Mediterranean region.

Another critical factor is that some of the seemingly early finds of opium poppy seeds outside of *P. setigerum* natural zone are not dated. Early deposits found in Israel^37^ but also in central Europe^38,39^ lack radiocarbon dates on the seeds or on direct contexts where the seeds were found. New efforts on dating these seeds and their contexts should be made before interpreting the route of cultivation/domestication^5,8^. Future studies on poppy seeds should integrate the morphometric as well as the direct dating approaches^36^. Likewise, it is foreseen to attempt to obtain aDNA from the archaeological seeds and so confirm, if possible, their status as domestic or cultivated.

## Conclusions

The present paper provides the first results of geometric morphometrics for *Papaver* taxa. The combination of descriptors such as the number of cells, size and shape of different modern species of *Papaver* allows to classify the seeds with good accuracy despite the methodological challenge due to the small size and globoid shape of poppy seeds. The classification model from the modern species used to assign archaeological seeds recovered at the late Neolithic site of Zurich-Parkhaus Opéra was also successful as it did attribute them to either *P. setigerum* and *P. somniferum.* The seeds were actually distributed within these two subspecies in equal parts, which might suggest that the plant has not yet acquired the morphometric characteristics of modern domestic seed. Further studies should be done in order to test the classification model. Future research should consider the study of opium poppy seeds from historical periods to confirm their assignation to the domesticated subspecies, as well as the study of earlier Neolithic finds in the Western Mediterranean in order to trace the pace of the domestication process.

## Methods

### Archeological material

One archaeological case included in the AgriChange Project^40^ was used in this paper: Zurich-Parkhaus Opéra (ZHOPE) located in Zurich, Switzerland is a Neolithic lake-dwelling site. A total of 33 whole and well-preserved uncharred waterlogged seeds (with visible cells) identified as *P*. *somniferum*^41^ were used. All seeds were obtained from the sample 12015.1B in layer 13 dated by dendrochronology between 3176 and 3153 BC (middle Horgen Culture^42^). Zurich-Parkhaus Opéra was excavated during the construction of subterranean parking in 2010 and 2011. Located in the northern shore of the Lake Zurich, eight settlement phases were identified and dendro-dated to 3234–2727 BC. In this late Neolithic site, archaeological deposits related to pile-dwelling houses are preserved in a waterlogged state where thousands of plant remains are present in charred and uncharred states, especially in layer 13, large quantities of opium poppy seeds were found concentrated mostly within building limits^41^.

These analyses were non-destructive and therefore no special permissions were required. Permission for the use of the archaeological seeds of Parkhaus Opéra for this study was granted by the scientific director of the project, Dr. Niels Bleicher (Office for Urbanism Zurich). Permission for the use of modern seed reference material was granted by the Graineterie of the National Museum of Natural History (MNHN) and no permission was necessary for the use of our own seed collection of the Integrative Prähistorische und Naturwissenschaftliche Archäologie (IPNA/IPAS).

### Data collection

**Figure 7:**
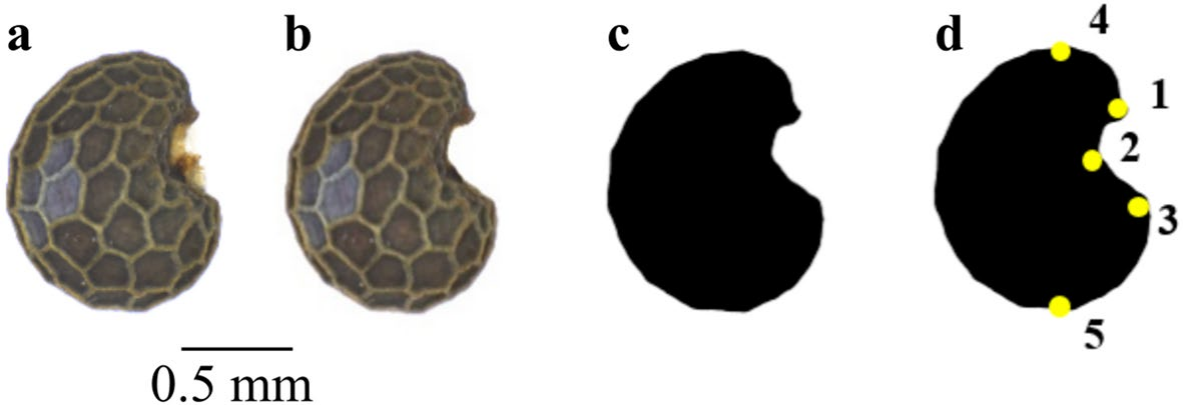
Data acquisition and post-processing (a) Lateral view position; (b) Background removal and cleaning; (c) Mask; (d) Landmarked-mask.

All *Papaver* seeds were photographed from a lateral view, with the hilum to the right. In this angle, it shows the cells, including those close to the hilum (Fig. 7A). The broader part of the seed at the bottom. The background of the seeds was a white surface to ease further background removal. The photos were made using Leica Z16 APO Binocular Stereo Microscope with a digital camera Leica DFC 420 and Leica Application Suite software (LAS 4.0, Leica), creating one mounted photo from several single photos that are stacked together to give depth of field to the seed and enable the counting of the number of seed cells. The background of the photo was removed manually using Photoshop 6 (Adobe) as well as the yellow soft tissue on the hilum part, in both archaeological and modern individuals (Fig. 7B). Then a mask (a black shape over a white background) was created using Photoshop (Fig. 7C). In order to normalise the outlines before elliptic Fourier transforms (EFT), coordinates of five landmarks were recorded using ImageJ^43^. The position of the landmarks was chosen in order to be the most reproducible as possible: two landmarks were positioned at the top and bottom extremes of the seeds and three around the hilum part (Fig. 7D). The landmark points covered most critical biological traits, from seed length (ldk: 4–5) to the hilum arch (ldk:1–3).

### Outline analysis

Seed shape was analysed using outline analysis based on elliptic Fourier transforms (EFT) using Momocs 1.3.0^44^ in a R 4.0.0 environment^45^. The elliptic Fourier transforms is a progressive decomposition of the outline (x; y) coordinates into a series of trigonometric functions called harmonics, associated with coefficients, used as quantitative shape variables. Here, outlines were normalised for their position, size and orientation using full generalised Procrustes alignment^46^. Landmark n°2 was used as the initial point for each outline. Then EFT was calculated from 360 points equally spaced along the curvilinear abscissa, and two landmarks (4 and 5) were extracted on each image. Based on harmonic power, five harmonics were retained and gathered 95% of the total harmonic power; more details on EFT can be found in Bonhomme et al*.*^44^.

### Measurement error

The poppy seeds are small and round and thus difficult to position in a specific orientation under the stereomicroscope. To minimise the error and aid with its reproducibility, some precautions were taken: the use of the same protocol, the same equipment, a single operator (RS) took the photos, a single operator (AJ) did all the cleaning and landmarking. As a preliminary step, all measurements were tested for the overall reproducibility. We used analyses of variance (ANOVA) following Claude^34^. The percentage of measurement error (%ME) is defined as “the ratio of the within measurement component of variance on the sum of the within- and among-measurement component”^34^. A set of five photos from three different taxa from the *P. somniferum* group (*P. setigerum*, *P. somniferum* and *P. nigrum*) were used in three different tests. The position test compares five photos of the same 15 seeds of the three different taxa by one single operator (RS). The cleaning and landmarking tests compared the repetition of the same action of digitalising cleaning and landmarks on the same photos (same 15 photos, same three species, three times).

### Phenotypic variation among species

The size (length and width of the bounding box) of the seed was recorded using the rectangular tool in ImageJ. The number of cells was counted for every seed using the multi-point tool in Image J. Length and width of the seeds were log-transformed^47,48^. Distributions of seed lengths, widths and cell number values were illustrated using boxplots. For each univariate variable (length measurements, cells number), overall differences were tested using Kruskal–Wallis non-parametric rank tests for multi-group comparison and Wilcoxon’s tests between each pairs of species.

To explore the overall shape variability, we used a principal component analysis (PCA) on the full matrix of Fourier coefficients and added the archaeological seeds as supplementary individuals. The first two principal components (see Results) were used as synthetic shape variables.

Then we used the coefficients on the first five harmonics in a permutational MANOVA using the package vegan^49^, to test for differences between taxa. A hierarchical clustering using UPGMA on the euclidean distance matrix between coefficients averaged per taxa is presented as an unrooted tree obtained with the package ape^50^.

To benchmark the performance of the different descriptors (width, length, number of cells, shape) at identifying species, we used linear discriminant analyses (LDA) provided by the package MASS^51^. Different combinations were used: first to all modern species, then only to *P. somniferum* group (*P*. *setigerum*, *P*. *somniferum* and *P*. *nigrum*) and finally only to *P. setigerum* and *P. somniferum*. To cope with unbalanced group sizes between sets due to the repeated *P. setigerum* and *P. somniferum*), we used random sampling of the over-represented classes so that they all sum up to 30. The process was repeated for 1000 permutations^52,53^. To compare the model performances with those obtained with chance alone, we also randomised labels and provide the maximum class accuracies (expected to follow a multinomial distribution), obtained among the permutations. The accuracies presented are the percentages of specimens correctly classified by using leave-one-out cross-validation. To visualise mean species shapes, we averaged Fourier coefficients and reconstructed seed outlines for each taxon.

### Archaeological identification

Each archaeological seed was classified using the “predictive” linear discriminant analyses trained on the modern material. For each seed, the dominant classification obtained along the 100 permutations was considered as the predicted class. The archaeological seeds were classified within three taxa of *P*. *somniferum: P. nigrum P*. *setigerum* and *P. somniferum* first, and after only classified within *P*. *setigerum* or *P. somniferum.* All descriptors (length, width, shape and number of cells) were used.

## Supplementary information

Supplementary Information.
