# Supplementary material for: A morphometric approach to track opium poppy domestication
Source: Sci Rep. 2021 May 7;11:9778. doi: 10.1038/s41598-021-88964-4 (PMC8105347; doi:10.1038/s41598-021-88964-4)
Supplement: Supplementary file 1 — Supplementary Information. [file 41598_2021_88964_MOESM1_ESM.docx]

**A morphometric approach to track opium poppy domestication**

Ana Jesus^1^, Vincent Bonhomme^2^, Allowen Evin^2^, Sarah Ivorra^2^, Raül Soteras^1^, Aurélie Salavert^3^, Ferran Antolín^1 4*^, Laurent Bouby^2^

**Supplementary information**

**Table 1**. List of Modern reference material used in this paper

| *Papaver* material | Status | Native | Species code | Supposed origin | Herbarium |
| --- | --- | --- | --- | --- | --- |
| *Papaver setigerum* ssp. *setigerum* | wild | Western Mediterranean | Pset.CSIBUB | Kanton Basel-Stadt | Collection Seminum Instituti Botanici Universitatis Basiliensis |
| *Papaver somniferum* L. ssp. *somniferum* com. *somniferum* var. *nigrum* Heyne | Dom. | unknown | Pnig.CSIBUB | Basel, Kanton Basel-Stadt | Collection Seminum Instituti Botanici Universitatis Basiliensis |
| *Papaver somniferum* L. ssp. *somniferum* | Dom. | unknown | Pset. CSIBUB | Porrentruy, Jura, Switzerland | Collection Seminum Instituti Botanici Universitatis Basiliensis |
| *Papaver setigerum* ssp. *setigerum* | wild | Western Mediterranean | Pset.GRA | Graineterie  (National Museum of Natural History, Paris) | Graneterie (National Museum of Natural History) |
| *Papaver somniferum* | Dom. | unknown | Psom.GRA | Graineterie  (National Museum of Natural History, Paris) | Graneterie (National Museum of Natural History) |
| *Papaver rhoeas* L. | wild | Temperate Eurasia | Prho. CSIBUB | Salzburg, Bergheim Tennisplatz ruderal Austria | Collection Seminum Instituti Botanici Universitatis Basiliensis |
| *Papaver hybridum* L. | wild | Southern Europe | Phyb. CSIBUB | Karlsruhe, Germany | Collection Seminum Instituti Botanici Universitatis Basiliensis |
| *Papaver argemone* L. | wild | Southern Europe | Parg. CSIBUB | Tübingen, Germany | Collection Seminum Instituti Botanici Universitatis Basiliensis |
| *Papaver dubium* L. | wild | Mediterranean | Pdub. CSIBUB | Mund, Switzerland | Collection Seminum Instituti Botanici Universitatis Basiliensis |

*
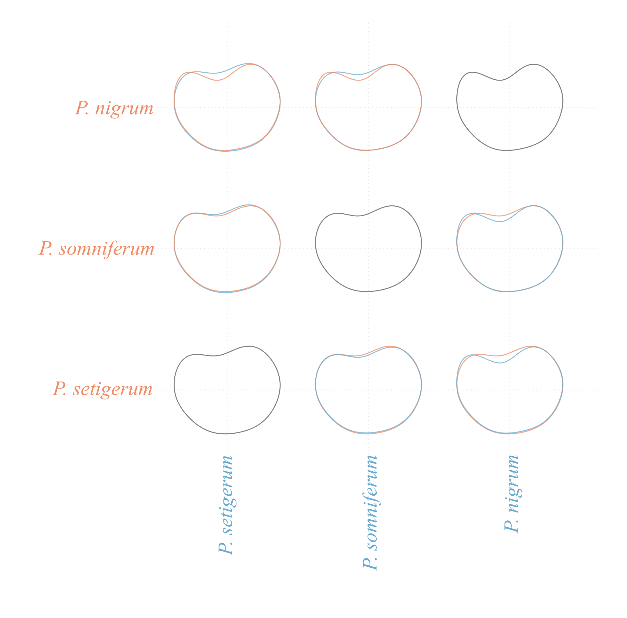
*

**Figure 1.** Mean shapes comparisons calculated for three subsp. of *P. somniferum.* Orange colour corresponds to the taxon of the rows and the blue colour to the taxon of the columns.


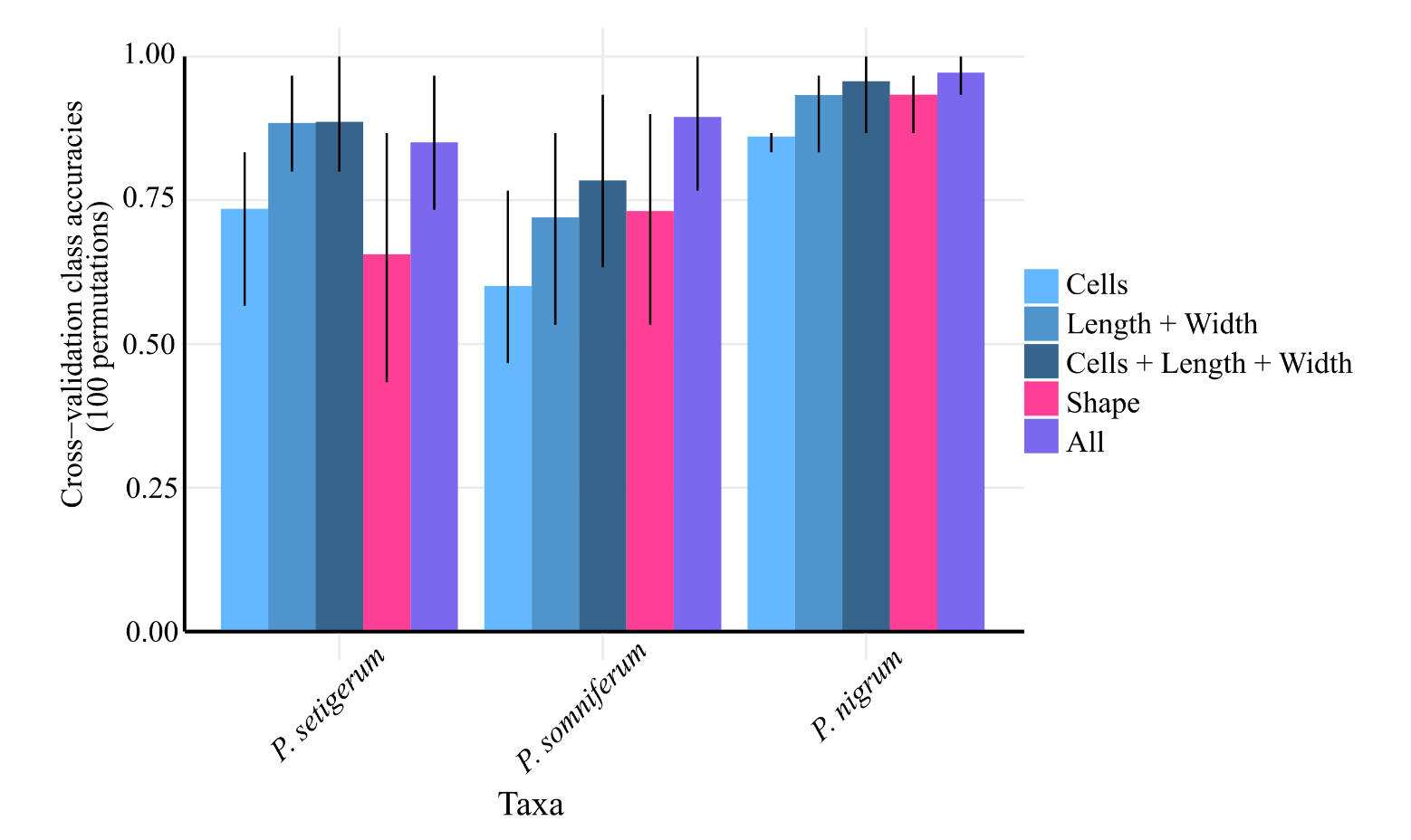


**Figure 2.** Benchmarking of linear discriminant analyses on three subspecies of *P. somniferum* and using different proxies. Accuracy per classes and their variability are being obtained using 100 permutations on classes-balanced dataset


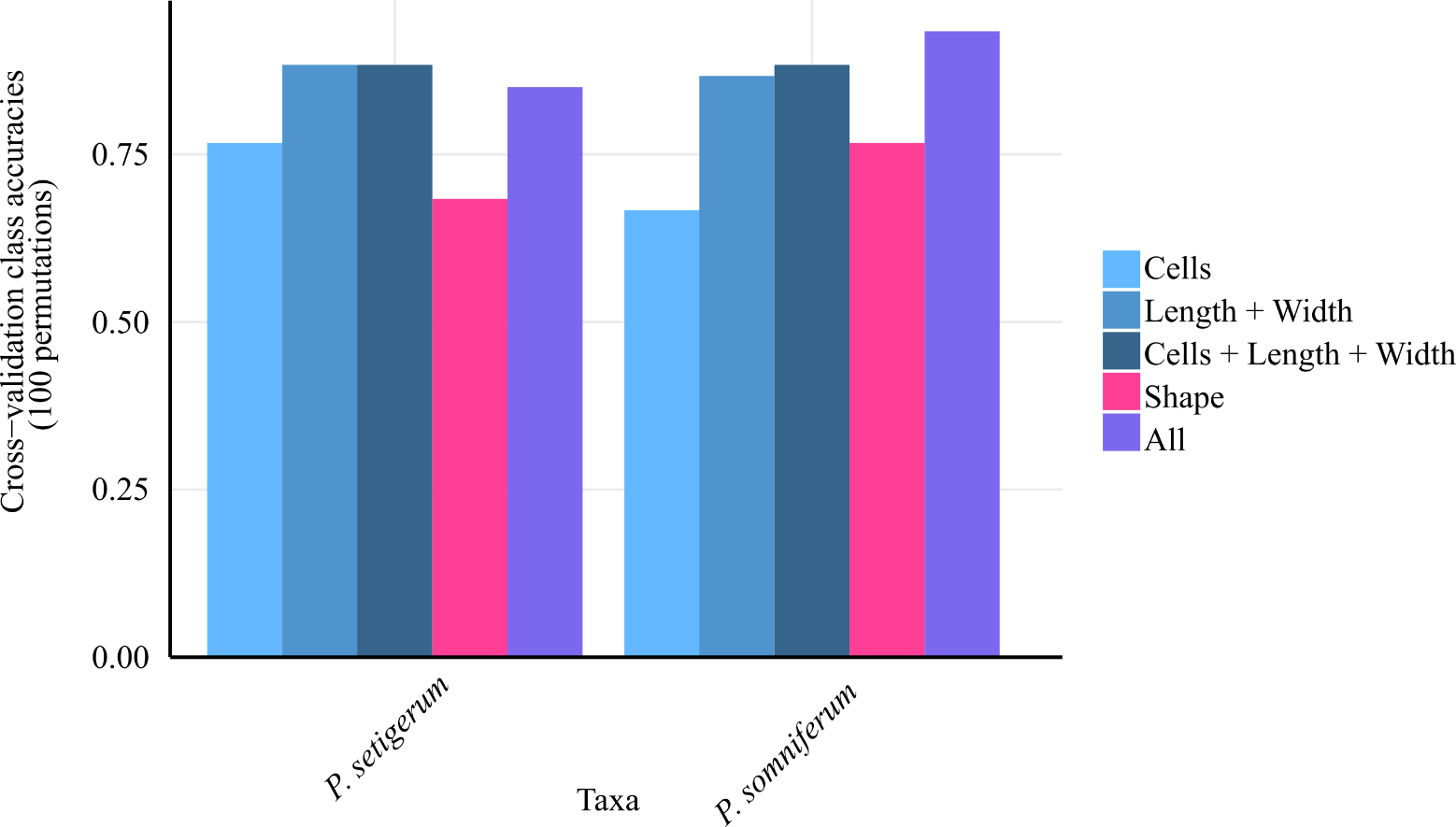


**Figure 3.** Benchmarking of linear discriminant analyses on two subspecies of *P. somniferum* and using different proxies. Accuracy per classes and their variability are being obtained using 100 permutations on balanced dataset, since they are balanced no need for error bars.
